# Hepatitis B virus and hepatitis D virus infection in women with or at risk for HIV infection in the United States

**DOI:** 10.3389/fmed.2023.1070420

**Published:** 2023-03-02

**Authors:** Ilona Argirion, Parag Mahale, Ruth M. Pfeiffer, Ping Liu, Adaora A. Adimora, Matthew J. Akiyama, Hector H. Bolivar, Audrey French, Michael Plankey, Jennifer C. Price, Aadia Rana, Anandi Sheth, Jill Koshiol, Eric C. Seaberg, Mark H. Kuniholm, Jeffrey Glenn, Thomas R. O’Brien

**Affiliations:** ^1^Infections and Immunoepidemiology Branch, Division of Cancer Epidemiology and Genetics, National Cancer Institute, Bethesda, MD, United States; ^2^Biostatistics Branch, Division of Cancer Epidemiology and Genetics, National Cancer Institute, Bethesda, MD, United States; ^3^Department of Medicine, Division of Gastroenterology and Hepatology, Stanford University School of Medicine, Palo Alto, CA, United States; ^4^School of Medicine and University of North Carolina Gillings School of Global Public Health, University of North Carolina at Chapel Hill, Chapel Hill, NC, United States; ^5^Division of General Internal Medicine, Department of Medicine, Albert Einstein College of Medicine, Montefiore Health System, Bronx, NY, United States; ^6^Division of Infectious Diseases, Department of Medicine, Albert Einstein College of Medicine, Montefiore Health System, Bronx, NY, United States; ^7^Department of Medicine, University of Miami Health System, Miami, FL, United States; ^8^Division of Neurology, Cook County Health, Chicago, IL, United States; ^9^Cook County Health, Hektoen Institute of Medicine, Chicago, IL, United States; ^10^Department of Medicine, Division of Infectious Diseases, Georgetown University Medical Center, Washington, DC, United States; ^11^Department of Medicine, University of California, San Francisco, San Francisco, CA, United States; ^12^Division of Infectious Diseases, University of Alabama-Birmingham Heersink School of Medicine, Birmingham, AL, United States; ^13^Division of Infectious Diseases, Emory University School of Medicine, Atlanta, GA, United States; ^14^Grady Health System, Infectious Diseases Program, Atlanta, GA, United States; ^15^Department of Epidemiology, Johns Hopkins Bloomberg School of Public Health, Baltimore, MD, United States; ^16^Department of Epidemiology and Biostatistics, University at Albany, State University of New York, Rensselaer, NY, United States

**Keywords:** epidemiology, hepatitis B virus, hepatitis D virus, hepatitis C virus, anti-HDV, persons who injected drugs

## Abstract

Hepatitis D virus (HDV) requires co-infection with hepatitis B virus (HBV). Human immunodeficiency virus (HIV) shares transmission routes with these viruses. Among 4,932 US women infected with or at-risk for HIV during 1994–2015, HBV surface antigen (HBsAg) positivity was more common in women with HIV (2.8% vs. 1.2%; *p* = 0.001); HDV was more common among participants enrolled during 2013–2015 (*p* = 0.0004) and those with resolved rather than active hepatitis C (1.9% vs. 0.5%; *p* = 0.02). Among HBsAg-positive women (*n* = 117), HDV antibody prevalence was 22% and did not vary by HIV status; HDV infection was associated with the presence of advanced fibrosis/cirrhosis at enrollment (adjusted odds ratio, 5.70; 95% confidence interval, 1.46–22.29). Our results demonstrate the importance of HDV testing in HBV-infected US women.

## Background

Hepatitis D virus (HDV) is a defective subviral pathogen that requires hepatitis B virus (HBV) for replication. An estimated 357,000 people in the United States are believed to have had past or ongoing HDV infection, although prevalence data are limited due to a paucity of previous studies (1).

HBV/HDV coinfected individuals experience more rapid progression to liver cirrhosis, hepatic decompensation, hepatocellular carcinoma, and death compared to those infected with HBV alone (2). HBV and HDV can be transmitted both sexually and through parenteral exposure. Given shared routes of transmission with human immunodeficiency virus (HIV) infection, people living with HIV (PLWH) or at risk for HIV infection are at higher risk for acquiring HDV. Furthermore, HDV-related liver disease may progress faster in PLWH, significantly impacting quality of life and survival (3). However, little is known about the prevalence of HDV infection in this population in the United States (4). With new, effective treatments for HDV potentially on the horizon (5, 6), it is important to determine HDV prevalence in high-risk populations, such as PLWH or at risk for acquiring HIV infection, to inform HDV screening and treatment.

The Women’s Interagency HIV Study (WIHS) is a large prospective study designed to investigate the treatment and prevention of HIV infection among US women. Using the HDV quantitative microarray antibody capture (Q-MAC) assay, we examined the prevalence of HDV infection among the WIHS participants overall and in subgroups defined by demographic, behavioral and clinical characteristics, including HIV infection status.

## Methods

### Study population

WIHS is a multi-center cohort study that was established in 1994 to investigate the natural history and treatment of HIV infection and associated morbidities among women living in the United States. Details regarding study methods and follow-up protocols have been previously described (7). WIHS investigators recruited 3,677 HIV-seropositive women and 1,305 sociodemographically similar HIV-seronegative women at 10 U.S. study sites over 4 enrollment waves between 1994 and 2015. Data are collected prospectively through semiannual physical examinations, biological specimen collections and structured interviewer-administered questionnaires to obtain information regarding sociodemographic and risk behaviors including whether the participant had ever injected drugs or participated in transactional sex (defined as exchange of sex for drugs, money, or shelter). In 2019, the WIHS became part of the MACS/WIHS Combined Cohort Study (MWCCS) with most women continuing enrollment under a similar protocol (8).

### Laboratory methods

Testing was performed on blood specimens collected at “baseline” (study entry for 99.8% of participants). As previously described (9), assays from Abbott Laboratories were used to detect hepatitis B surface antigen (HBsAg; Auszyme Microparticle enzyme immunoassay [EIA]). We considered women who tested positive for HBsAg at baseline to have “active HBV infection” at that time point. In these participants, we used HBsAg test results from subsequent visits, performed using the Seimens ADVIA Centaur Immunoassay System, and classified “chronically infected” participants as those with a second positive HBsAg result at least 180 days after the initial positive result.

Hepatitis C (HCV) antibody status was assessed in baseline samples using enzyme immunoassay (EIA) 2.0 [Abbott Laboratories] and 3.0 [Ortho-Clinical Diagnostics] with testing for HCV RNA using either the COBAS Amplicor HCV Detection Kit, COBAS Taqman Assay [Roche Diagnostics] or Quantiplex 2.0 branched chain DNA-enhanced label amplification assay [Bayer-Versant Diagnostics] in participants who were positive for HCV antibody. We defined HCV status as “never infected” for participants who tested negative for HCV antibodies, “resolved infection” for those who tested positive for anti-HCV but negative for HCV RNA and “actively infected” for those who tested positive for HCV RNA. HIV infection status was based on results of an enzyme-linked immunosorbent assay with western blot confirmation [NASBA/NuciSens HIV RNA assay, BioMerieux] (10).

Baseline measures of aspartate aminotransferase (AST) levels and platelet counts were determined using standard laboratory protocols. APRI was calculated as (100 x [AST/AST ULN]/platelet count [10^9^ /l]) (11).

Because HDV replication requires HBsAg, we limited testing for HDV to reposited baseline samples from participants with active HBV infection. As previously described (12), HDV Q-MAC is a high-throughput assay with high sensitivity and specificity for detecting anti-HDV immunoglobulin G (12, 13). After excluding two participants without an available specimen, we tested 117 HBsAg-positive participants for anti-HDV.

### Statistical analysis

Among women who were tested for HBsAg, we examined the prevalence of active HBV infection and the prevalence of HDV infection. We also determined anti-HDV seroprevalence among participants with active HBV infection. To assess whether infection prevalence differed in subgroups, we examined prevalence in strata defined by demographic, behavioral and clinical characteristics. Prevalence estimates were compared by *χ*^2^ test or Fisher’s exact test (when ≥25% of cells had a count <5).

Unconditional logistic regression was used the assess the association between HDV infection and selected variables, including year of enrollment, transactional sex, HCV status, injection drug use, and HIV infection. Multivariable analyses were not possible due to sparse data for some of those variables.

Polytomous logistic regression models were used to estimate the odds ratio (OR) and 95% confidence interval (CI) for associations between HDV infection and the aspartate aminotransferase to platelet ratio index (APRI). Measures of APRI were categorized in the following manner: no significant fibrosis (<0.5), significant fibrosis (0.5 to <1.0), advanced fibrosis/cirrhosis (≥1.0). Age at baseline, HCV status (dichotomized as actively infected vs. never infected/resolved infection) and HIV status were considered as potential confounders.

All analyses were performed with SAS software version 9.4 (SAS Institute, Inc.)

## Results

Among the entire WIHS cohort of 4,982 women, 4,932 (99.0%) were tested for HBsAg at baseline. The median age at enrollment for the overall cohort was 36 years, 2,588 (52.5%) of participants were recruited between 1994 and 1995, and 3,033 (61.5%) of the women were non-Hispanic Black (Table 1). At baseline, 1295 (26.3%) reported ever having injected drugs, 1713 (34.7%) reported a history of transactional sex, 1,045 (21.2%) were actively infected with HCV, and 3,634 (73.7%) were HIV-positive. Women with active HBV infection (i.e., HBsAg-positive at baseline) represented 2.4% of the analytic cohort.

**Table 1:** Demographic, behavioral, and clinical characteristics of women screened for HBsAg at enrollment in the WIHS cohort.

Characteristic	Full Cohort^‡^	HBsAg+
Sample size, N (%)	4,932	117
Median Age (IQR), years	36 (30–43)	35 (30–40)
Year of Enrollment
1994–95	2,588 (52.5)	66 (56.4)
2001–02	1,142 (23.2)	16 (13.7)
2011–12	356 (7.2)	5 (4.3)
2013–15	846 (17.2)	30 (25.6)
Race/Ethnicity, N (%)
White (non-Hispanic)	658 (13.3)	12 (10.3)
White (Hispanic)	348 (7.1)	5 (4.3)
Black (non-Hispanic)	3,033 (61.5)	87 (74.4)
Black (Hispanic)	110 (2.2)	2 (1.7)
Other (Hispanic)	625 (12.7)	10 (8.5)
Asian/Pacific Islander	49 (1.0)	0 (0.0)
Native American/Alaskan Native	37 (0.8)	1 (0.9)
Other	72 (1.5)	0 (0.0)
Country of Birth
United States	3,953 (80.2)	98 (83.8)
Other	947 (19.2)	14 (12.0)
Missing	32 (0.6)	5 (4.3)
Injection Drug Use, N (%)
Yes	1,295 (26.3)	36 (30.8)
No	3,637 (73.7)	81 (69.2)
Transactional sex, N (%)
Yes	1713 (34.7)	56 (47.9)
No	3,205 (65.0)	61 (52.1)
Missing	14 (0.28)	0 (0.0)
HBsAg, N (%)
Positive	117 (2.4)	117 (100%)
Negative	4,813 (97.6)	0 (0%)
HCV Status, N (%)
Active	1,045 (21.2)	20 (17.1)
Resolved	367 (7.4)	19 (16.2)
Never Infected	3,512 (71.2)	78 (66.7)
Missing	8 (0.2)	0 (0.0)
HIV, N (%)
Positive	3,634 (73.7)	102 (87.2)
Negative	1,298 (26.3)	15 (12.8)

HCV, hepatitis C virus; HIV, human immunodeficiency virus; HBsAb, hepatitis B surface antibody; IQR, interquartile range.

‡ 2 HBsAg + participants were excluded from analysis due to lack of serum for HDV testing.

We examined the prevalence of active HBV infection, by demographic, behavioral and clinical characteristics (Supplemental Table 1). HBV infection was more common in women who enrolled in 1994–95 (66 [2.6%]) or 2013–15 (30 [3.6%]) than those who entered the study during 2001–02 (16 [1.4%]) or 2011–12 (5 [1.4%]). HBV infection was not significantly more common in women who reported injection drug use (36 [2.8%] vs. 81 [2.2%]; *p* = 0.27) but was found more often in women who reported engaging in transactional sex (56 [3.3%]) than those who did not (61 [1.9%]; *p* = 0.004). Active HBV infection was more common in women with resolved HCV infection (19 [5.2%]) than those with either active HCV infection (20 [1.9%]) or those who had never been infected (78 [2.2%]). Active HBV was present in 102 (2.8%) of women with HIV infection compared to 15 (1.2%) of uninfected women (*p* = 0.001). 85 (72.6%) participants had subsequent testing for HBsAg (median time to repeat testing, 6.9 years). The repeat test was positive for 47 (55.3%), who presumably had chronic hepatitis B, and negative for 38 (44.7%), who could have had acute HBV infection at study entry or chronic infection followed by loss of HBsAg either spontaneously or due to treatment.

Among WIHS participants overall, 26/4932 (0.5%) were positive for anti-HDV (Table 2). Anti-HDV prevalence did not differ by age, but participants who entered the cohort in 2013–2015 were more likely to be anti-HDV positive compared to those who enrolled in 1994–95 (13 [1.5%] vs. 10 [0.4%]; *p* = 0.0004). With the exception of one participant whose country of birth was missing, all anti-HDV seropositive participants were born in the United States. Women who reported injection drug use or transactional sex had a higher prevalence of anti-HDV seropositivity, although those differences were not statistically significant. Compared to the women who enrolled in 1994–1995, the participants who enrolled in 2013–2015 were less likely to report injection drug use (61 [7.2%] vs. 1,034 [40.0%]) and equally likely to report transactional sex (314 [37.1%] vs. 951 [36.9%]). Women with resolved HCV infection were approximately four times more likely to have anti-HDV seropositivity than those with active HCV infection (7 [1.9%] vs. 5 [0.5%], respectively; *p* = 0.02). There was no significant difference in HDV-seropositivity between those who were never infected with HCV and those with active HCV infection (14 [0.4%] vs. 5 [0.5%], respectively; *p* = 0.78). The prevalence of anti-HDV seropositivity was 0.6% (95% CI: 0.4–0.9) among women with HIV infection and 0.4% (95% CI: 0.2–0.9) in those who were HIV negative.

**Table 2:** Prevalence of HDV antibody among WIHS participants, by selected demographic, behavioral and clinical characteristics.

Characteristics	Anti-HDV positive	All participants			Active HBV infection (HBsAg positive)		
		Total no.	Prevalence of anti-HDV, % (95%CI)^b^	*p*-Value^a^	Total no.	Prevalence of anti-HDV, % (95%CI)^b^	*p*-Value^a^
Overall	26	4,932	0.5 (0.4–0.8)	–	117	22.2 (15.6–30.6)	–
Age at enrollment, years^c^				0.52			0.16
16–29	4	1,123	0.4 (0.1–0.9)		27	14.8 (5.9–32.5)	
30–39	10	2026	0.5 (0.3–0.9)		57	17.5 (9.8–29.4)	
40–49	10	1,289	0.8 (0.4–1.4)		28	35.7 (20.7–54.2)	
≥50	2	494	0.4 (0.1–1.5)		5	40.0 (11.8–76.9)	
Race/Ethnicity, N (%)*							
White (non-Hispanic)	3	658	0.5 (0.2–1.3)	(ref)	12	25.0 (8.9–53.2)	(ref)
Black (non-Hispanic)	23	3,033	0.8 (0.5–1.1)	0.61	87	26.4 (18.3–36.6)	1.00
Country of Birth							
United States	25	947	2.6 (1.7–3.9)	–	98	25.5 (17.2–35.3)	–
Other	0	3,953	–	–	14	–	–
Year of Enrollment							
1994–95	10	2,588	0.4 (0.2–0.7)	(ref)	66	15.2 (8.4–25.7)	(ref)
2001–02	3	1,142	0.3 (0.1–0.8)	0.77	16	18.8 (6.6–43.0)	0.72
2011–12	0	356	–	–	5	–	–
2013–15	13	846	1.5 (0.9–2.6)	0.0004	30	43.3 (27.4–60.8)	0.02
Injection Drug Use							
Yes	10	1,295	0.8 (0.4–1.4)	0.16	36	27.8 (15.9–44.0)	0.45
No	16	3,637	0.4 (0.3–0.7)	(ref)	81	19.8 (12.5–29.7)	(ref)
Transactional Sex							
Yes	13	1713	0.8 (0.4–1.3)	0.15	56	23.2 (14.1–35.8)	0.84
No	13	3,205	0.4 (0.2–0.7)	(ref)	61	21.3 (12.9–33.1)	(ref)
HCV Status							
Active	5	1,045	0.5 (0.2–1.1)	(ref)	20	25.0 (8.7–49.1)	(ref)
Resolved	7	367	1.9 (0.8–3.9)	0.02	19	36.8 (16.3–61.6)	0.74
Never Infected	14	3,512	0.4 (0.2–0.7)	0.78	78	18.0 (10.2–28.3)	0.55
HIV							
Positive	21	3,634	0.6 (0.4–0.9)	0.41	102	20.6 (13.9–29.4)	0.37
Negative	5	1,298	0.4 (0.2–0.9)	(ref)	15	33.3 (15.2–58.3)	(ref)

^*^HDV infection was detected only in women of White (non-Hispanic) or Black (non-Hispanic) “Race/Ethnicity”.

CI, confidence interval; HCV, hepatitis C virus; HDV, hepatitis D virus; HIV, human immunodeficiency virus; HBsAg, hepatitis B surface antigen.

a *p*-value from chi-square test unless indicated otherwise.

b Exact binomial CIs.

c *p*-value for trend from univariate logistic regression for each category increase of age.

Prevalence estimates among subgroups were compared by Chi-squared test or Fisher’s exact test, as appropriate. All tests were two-sided and *p*-values < 0.05 were considered statistically significant.

Among the 117 women with active HBV infection who were tested for anti-HDV, 26 (22.2%) were positive for HDV antibody (Table 2). HDV prevalence increased with age at enrollment. While a test for trend in age categories was not significant, median age was 39.0 years for HDV-positive women and 34.0 years for HDV-negative women (*p* = 0.02). As per the overall cohort, among women with active HBV infection, anti-HDV seropositivity was significantly higher in those who enrolled in 2013–2015 (13 [43.3%]) than those who enrolled in 1994–95 (10 [15.2%]; p = 0.02; OR, 4.3; 95% CI, 1.6–11.5; Supplemental Table 2). Anti-HDV seropositivity prevalence was higher in women who reported injection drug use (10 [27.8%]) than those that did not (16 [19.8%]), but that difference was not statistically significant (*p* = 0.45). Among women with active HBV infection, antibody to HDV was present in 21 (20.6%) of the women with HIV infection, compared to 5 (33.3%) who were not infected with HIV (*p* = 0.37).

We examined the relationship between HDV infection, and the presence of fibrosis as defined by APRI-based categories among the participants with active HBV. Compared to women who had no significant fibrosis, those with advanced fibrosis/cirrhosis where much more likely to be positive for HDV, (OR = 6.00; 95%CI: 1.61–22.42) (Table 3). After adjusting for age at baseline, this association was slightly attenuated (adjusted OR = 5.70; 95%CI: 1.46–22.29). HCV status did not confound this relationship. We were unable to assess potential confounding by HIV status because all participants with advanced fibrosis/cirrhosis were HIV-positive. No association was found between HDV status and significant fibrosis when compared to no significant fibrosis (OR = 1.88; 95%CI: 0.62–5.63).

**Table 3:** Odds ratios (ORs) and 95% confidence intervals (CIs) for the association between HDV and fibrosis category (based on APRI values at enrollment) among WIHS participants with active HBV infection (*n* = 117).

	APRI		
	OR (95%CI)		*p*-value*
	Significant fibrosis vs. no significant fibrosis	Advanced fibrosis/cirrhosis vs. no significant fibrosis	
Unadjusted	1.88 (0.62, 5.63)	6.00 (1.61, 22.42)	0.03
Adjusted for age at baseline	1.72 (0.55, 5.33)	5.70 (1.46, 22.29)	0.04

^d^APRI categories: “no significant fibrosis”, <0.5 (*N* = 84); “significant fibrosis”, 0.5 to < 1.0 (*N* = 22); “advanced fibrosis/cirrhosis”, ≥1.0 (*N* = 11).

*value of ps calculated as *p*-trend.

## Discussion

We evaluated the prevalence of anti-HDV seropositivity in a cohort of US women with or at risk of HIV infection. Using an assay with excellent performance characteristics for detecting anti-HDV, 22% of women with active HBV infection had evidence of HDV infection. While the women with HIV had a significantly higher prevalence of active HBV infection, anti-HDV prevalence did not differ by HIV status, although power was limited.

While anti-HDV seroprevalence was common among HBsAg-positive women enrolled in WIHS, the prevalence was lower than that reported in previous studies. Among people who inject drugs enrolled in the Urban Health Study (UHS), we found that 35.6% of those with active HBV infection were positive by the HDV QMAC assay (13). Other studies of HBV/HDV co-infection among individuals in the US who inject drugs reported prevalence estimates from 42 to 67% (14–16). In the 2011–2016 National Health and Nutrition Examination Survey, which is based on the general US population, investigators reported anti-HDV prevalence was 42% in those with active HBV. (1) Reported differences in anti-HDV prevalence may reflect differences in population, associated risk factors and test characteristics of anti-HDV assays. A limitation of this study is the lack of data on HDV RNA; nevertheless, in a previous study conducted in the United States by our group, all participants who tested positive by QMAC was also found to be positive for HDV RNA (13).

Anti-HDV seroprevalence was highest among the women who enrolled during 2013–2015, which is the latest period of enrollment. However, that finding should be interpreted with caution because WIHS enrollment in 2013–2015 was almost exclusively from southern U.S. states, whereas participants from other regions predominated in earlier enrollment waves. The higher prevalence of anti-HDV among the most recent WIHS enrollees thus could reflect either temporality or geography. To our knowledge, prevalence by geographical region has not been assessed in national surveys of HDV infection in the United States (1) and might be the subject of future studies.

Interactions between hepatitis viruses affect viral clearance (13). In the cohort overall, active HBV infection was more common in WIHS participants with resolved hepatitis C than those with active HCV infection, or those who had never been infected with HCV. These results correspond with our observations in UHS (13). In the cohort overall, anti-HDV prevalence was lower in those with active HCV infection than in those with resolved HCV infection. Among HBsAg+ positive participants, there were no meaningful difference in HDV prevalence by HCV status, whereas in UHS, we saw a slightly higher HDV prevalence in HBsAg+ participants with resolved compared to chronic HCV infection (13). Due to the cross-sectional design of these studies, temporality of viral acquisition could not be assessed, nevertheless, these results suggest that active HBV suppressed HCV infection in these populations. Longitudinal studies are needed to clarify interactions among hepatitis viruses.

In accordance with previous studies (2, 17), we found an association between HDV status and advanced fibrosis/cirrhosis at enrollment. Although we were limited by our lack of data on duration of infection, adjusting for age at baseline as a proxy measure still yielded a significant association between HDV status and advanced fibrosis/cirrhosis as defined by APRI.

Current guidelines for HDV testing recommend that persons belonging to certain ‘high-risk’ groups (including those with HIV or HCV infection, persons who have ever injected drugs, individuals with multiple sex partners) who test positive for HBsAg should also be tested for HDV infection (18). If we consider a history of transactional sex as an indicator of multiple sex partners, 89.7% of the women who were positive for HBsAg and 88.4% of the women who were positive for HDV had at least one risk factors that should prompt clinical testing for HDV. Data on previous HDV testing in clinical practice is not available for WIHS participants.

Our results provide additional evidence that HDV is common among individuals with active HBV infection in the US and support current recommendations that persons belonging to ‘high-risk’ groups who test positive for HBsAg should be tested for HDV infection (18). Little is known about the uptake of HDV screening recommendations in the United States. Future efforts should evaluate HDV testing rates in the US and seek to raise awareness regarding the importance of screening for this highly pathogenic virus.

## Data availability statement

Access to individual-level data from the MACS/WIHS Combined Cohort Study Data (MWCCS) may be obtained upon review and approval of a MWCCS concept sheet. Links and instructions for online concept sheet submission are on the study website (https://statepi.jhsph.edu/mwccs/work-with-us/).

## Author contributions

IA, PM, RP, and TO’B contributed to the conception and design of the study. PL and JG conducted laboratory analyses. AA, MA, HB, AF, MP, JP, AR, AS, ES, and MK manage the cohort and corresponding data. IA conducted the statistical analyses and took the lead in writing the manuscript. All authors provided critical feedback and review of the manuscript.

## Funding

This work was supported by the Intramural Research Program of the National Institutes of Health, National Cancer Institute, Division of Cancer Epidemiology and Genetics. The MWCCS is funded primarily by the National Heart, Lung, and Blood Institute (NHLBI), with additional co-funding from the Eunice Kennedy Shriver National Institute Of Child Health & Human Development (NICHD), National Institute On Aging (NIA), National Institute of Dental & Craniofacial Research (NIDCR), National Institute of Allergy And Infectious Diseases (NIAID), National Institute of Neurological Disorders And Stroke (NINDS), National Institute of Mental Health (NIMH), National Institute On Drug Abuse (NIDA), National Institute Of Nursing Research (NINR), National Cancer Institute (NCI), National Institute on Alcohol Abuse and Alcoholism (NIAAA), National Institute on Deafness and Other Communication Disorders (NIDCD), National Institute of Diabetes and Digestive and Kidney Diseases (NIDDK), National Institute on Minority Health and Health Disparities (NIMHD), and in coordination and alignment with the research priorities of the National Institutes of Health, Office of AIDS Research (OAR). MWCCS data collection is also supported by UL1-TR000004 (UCSF CTSA), UL1-TR003098 (JHU ICTR), UL1-TR001881 (UCLA CTSI), P30-AI-050409 (Atlanta CFAR), P30-AI-073961 (Miami CFAR), P30-AI-050410 (UNC CFAR), P30-AI-027767 (UAB CFAR), and P30-MH-116867 (Miami CHARM). Additional support was provided by the Johns Hopkins University Center for AIDS Research (P30AI094189).

## Conflict of interest

The authors declare that the research was conducted in the absence of any commercial or financial relationships that could be construed as a potential conflict of interest.

## Publisher’s note

All claims expressed in this article are solely those of the authors and do not necessarily represent those of their affiliated organizations, or those of the publisher, the editors and the reviewers. Any product that may be evaluated in this article, or claim that may be made by its manufacturer, is not guaranteed or endorsed by the publisher.
